# “They’re Not Going to Do Nothing for Me”: Research Participants’ Attitudes towards Elective Genetic Counseling

**DOI:** 10.3390/jpm10040143

**Published:** 2020-09-24

**Authors:** Erica J. Sutton, Annika T. Beck, Kylie O. Gamm, Jennifer B. McCormick, Iftikhar J. Kullo, Richard R. Sharp

**Affiliations:** 1 Biomedical Ethics Research Program, Mayo Clinic, Rochester, MN 55905, USA; suttonericaj@gmail.com (E.J.S.); beck1477@umn.edu (A.T.B.); 2 Department of Health Sciences Research, Mayo Clinic, Rochester, MN 55905, USA; 3 Department of Psychiatry and Psychology, Mayo Clinic, Rochester, MN 55905, USA; osterhus.kylie@mayo.edu; 4 College of Medicine, Department of Humanities, Pennsylvania State University, Hershey, PA 17033, USA; jmccormick@pennstatehealth.psu.edu; 5 Division of Cardiovascular Diseases, Mayo Clinic, Rochester, MN 55905, USA; kullo.iftikhar@mayo.edu

**Keywords:** genomic sequencing, genetic counseling, informed consent, patient communication, return of results, ethical, legal, and social implications, patient education

## Abstract

As applications of genomic sequencing have expanded, offering genetic counseling support to all patients is arguably no longer practical. Additionally, whether individuals desire and value genetic counseling services for genomic screening is unclear. We offered elective genetic counseling to 5110 individuals prior to undergoing sequencing and 2310 participants who received neutral results to assess demand. A total of 0.2% of the study participants accessed genetic counseling services prior to sequencing, and 0.3% reached out after receiving neutral results. We later conducted 50 interviews with participants to understand why they did not access these services. Many interviewees did not recall the availability of genetic counseling and were unfamiliar with the profession. Interviewees described not needing counseling before sequencing because they understood the study and felt that they could cope with any result. Counseling was considered equally unnecessary after learning neutral results. Although the participants had questions about their results, they did not feel that speaking with a genetic counselor would be helpful. Genomic screening efforts that employ opt-in models of genetic counseling may need to clarify the potential value of genetic counseling support from the outset and feature genetic counseling services more prominently in program materials.

## 1. Introduction

The rapid expansion of genomic sequencing has required adaptations to traditional models of in-person genetic counseling to allow genetic specialists to focus their time on the most complex cases [1,2,3]. Innovative approaches to genetic counseling include the expanded use of telephone counseling [1,4], telegenetics [5,6], interactive websites and chatbots [3,7], as well as combinations of different mediums [8]. To make the provision of genetic counseling services for genomic sequencing feasible, The Mayo Clinic’s Return of Actionable Variants Empirical (RAVE) study offered complimentary elective genetic counseling prior to sequencing and upon receiving neutral results (i.e., no variants of clinical significance), and provided in-person genetic counseling to individuals who received positive sequencing results [9]. Inviting upwards of 5000 individuals to participate in the RAVE study and sequencing 2500 participants’ samples required that we reimagine the delivery of genetic counseling services. Making genetic counseling optional allowed us to assess the demand for these services at two distinct time points: prior to undergoing sequencing and after receiving neutral results. We were surprised how few participants (0.2%) took advantage of this free resource prior to undergoing sequencing [9] and, as we report in this manuscript, how few participants accessed these resources after receiving their results.

A low uptake of genetic counseling services is common, not only in clinical and research settings, but also in direct-to-consumer contexts [7,9,10,11]. Potential explanations for the underutilization of genetic counseling resources include patients and research participants not having a good understanding of the role of a genetic counselor, a lack of clarity about how they might benefit from speaking with a counselor, not being worried about their genetic screen or test result, and a belief that they have understood their results [7,10,11].

Studies examining patient and research participant attitudes about genetic counseling are scant [11,12,13,14]. Having observed what we considered to be a significant underutilization of genetic counseling services in the context of the RAVE study, we sought to characterize this phenomenon more fully. Here, we share our qualitative findings on the lack of demand for genetic counseling prior to sequencing and upon the return of neutral results. Patient and research participant attitudes about genetic counseling and its value will play a significant role in individuals’ experiences of new applications of genomic sequencing technologies. Therefore, patient and research participants’ perspectives should be examined and incorporated into the design of innovative ways to provide optimum genetics care.

## 2. Materials and Methods

### 2.1. Ethics Statement

This study was approved by the Mayo Clinic Institutional Review Board (IRB#: 15-005013).

### 2.2. Targeted Genomic Sequencing Study: Assessing Demand for Genetic Counseling

Our inquiry occurred within the Return of Actionable Variants Empirical (RAVE) study, part of a National Institutes of Health (USA) funded consortium that reported pathogenic/likely pathogenic results in 68 disease-related genes and 14 single-nucleotide variants [15]. A total of 5110 contributors from two Mayo Clinic biorepositories were invited by mail to participate. Invitees had high cholesterol, high triglycerides, and/or colon polyps. To enroll, they had to agree to receive genetic results related to these health conditions and have them placed in their electronic health record. The size of the RAVE study, coupled with limited genetic counseling resources, made speaking with each participant in advance of enrollment impossible. Similarly, returning all neutral results in-person or by telephone to thousands of participants was not feasible. Consequently, participant-initiated, complimentary genetic counseling was offered, but not required. Study invitees received a 14-page consent document, a 4-page informational brochure, and a 2-page frequently asked questions (FAQ) handout in the mail. The availability of optional genetic counseling services was mentioned in all of these materials. Of the 5110 individuals invited to participate in the RAVE study, eight (0.2%) spoke with a genetic counselor prior to undergoing sequencing [9].

Of the 2535 individuals who underwent genomic sequencing as part of the RAVE study, 2310 participants received neutral genomic sequencing results in the mail. The mailed materials consisted of a one-page results letter and a laboratory report. The letter reminded participants that they had participated in the RAVE study and that their genetic material had been sequenced. In bold, underlined text in the top third of the letter, they were informed, “Your results: No clinically significant genetic variants were found. No further action on your part is needed at this time.” The letter also described several test limitations and, at the end, invited participants to call a genetic counselor with any questions. The clinical laboratory report presented participants’ sequencing results in more technical language and included several results tables.

### 2.3. Interview Study Recruitment

We recruited participants for our qualitative inquiry from the first 300 participants who received neutral results, which were randomly returned in batches of 50. Consequently, we were limited in terms of the purposive sampling we could conduct. We recruited the first 30 individuals who agreed to participate; for the remaining 20 participants, we oversampled males, younger participants, and individuals whose baseline survey at the time of study enrollment indicated lower genetic knowledge scores [16]. We recruited individuals approximately 2 weeks after the mail-out of their results packet and conducted telephone interviews, on average, 25 days after the mail-out (range: 19 to 43 days). We did not ask individuals who consented to participate in an interview to find or review their results letter in advance of the interview, nor did we ask them to have it accessible as a point of reference during the interview. Our objective was to ascertain how participants had understood their results and what actions they took in response to those results.

### 2.4. Data Collection

To assess the demand for genetic counseling following the return of neutral results, genetic counselors completed field notes to document how many recipients reached out to them and the nature of callers’ questions.

Qualitative interviews focused on participants’ overall experience in the RAVE study, receiving neutral genomic sequencing results by mail, and their reactions to those results. To gain a better understanding of why so few people accessed genetic counseling services, we asked a series of questions about the study’s offer to speak with a genetic counselor before enrollment and upon receiving results, whether participants were familiar with genetic counseling, and why participants chose not to access genetic counseling services during their study participation.

All the telephone interviews were conducted by E.J.S. or A.T.B. Interviews were audio-recorded and transcribed verbatim. Transcripts were reviewed for accuracy and edited accordingly. Transcript review also served as an opportunity for preliminary data analysis. Cleaned transcripts were uploaded into NVivo for data management [17].

### 2.5. Data Analysis

We exported all the transcript text that mentioned genetic counseling, genetic counseling services, genetic counselors, or the genetic counseling profession. We included all interview guide questions and probes related to genetic counseling and associated participant responses. Text featuring participant questions of the interviewers were also examined. Three independent coders (E.J.S., A.T.B., K.O.G.) participated in codebook development and coding.

We began codebook development from 85 pages of transcript excerpts from 19 interviews and transcription analysis notes. We then implemented a three-pronged approach to codebook refinement: first, we applied the codebook to six transcripts taken longitudinally over the course of the study, appreciating the evolution of the interviews over time; second, we applied the modified codebook to five of the most challenging interviews to make sure the codebook was effective in capturing the content of more difficult interviews; third, we applied the codebook to five interviews near the end of the study, three of which were conducted by a second interviewer. Consensus was reached between all three coders at every stage of codebook development. This iterative, comprehensive approach reduced the need for later adjustments to the codebook, as it was applied to the remaining transcripts.

All three coders participated in thematic analysis and memo writing [18]. We identified themes related to understandings of the RAVE study, understandings of the genetic counseling profession, and the perceived value (or lack thereof) of genetic counseling services. NVivo outputs were divided among EJS and ATB for detailed memo writing and drafting of thematic summaries.

## 3. Results

Of the 2310 study participants who received the neutral results packet in the mail, six spoke with a genetic counselor (0.3%). Of the six participants who called, three wanted to discuss their family history of Alzheimer’s disease (a condition not examined as part of the RAVE study), two wanted to confirm that they had understood their results, and one was confused by her results and wanted them explained.

We invited, by telephone, 123 RAVE participants, who had been among the first to receive their neutral results by mail, to participate in an interview. Fifty-five individuals agreed to participate; 50 completed the interview (see Table 1).

We spoke to equal numbers of female and male participants who ranged in age from 42 to 71 years. They were predominantly white. All had a high school education and most had some college. All the participants had some form of health insurance, and most reported a comfortable income. The participants offered a number of explanations as to why they did not access elective genetic counseling either before they enrolled in the RAVE study or after receiving neutral genomic sequencing results by mail (see Box 1).

Box 1 **Common explanations for not accessing elective genetic counseling services prior to sequencing or after receiving neutral results.**

- Participants did not recall that genetic counseling had been offered.
- Participants were unfamiliar with the genetic counseling profession and associated services.
- Participants believed that genetic counseling was for other people, particularly individuals who receive positive results.
- Participants were committed to research and understood the risks of study participation.
- Participants did not know how a genetic counselor could help them before or after screening.

### 3.1. Recalling the Availability of Genetic Counseling Service

The majority of participants did not remember that genetic counseling had been an option prior to undergoing sequencing. More interviewees recalled the availability of post-result genetic counseling services. While half of our participants had their results letter with them during the interview, many were still not aware that they could speak with someone. Participants who did not recall the availability of genetic counselors at different time points throughout the study were not surprised that such a service would have been provided: “[I]f you told me it was there, I believe you” (RNR 51); “[It’s] a reasonable thing to have available” (RNR 31). Some participants explained that while they did not access genetic counseling services, they found the offer reassuring.

### 3.2. Unfamiliar with the Genetic Counseling Profession

Most participants lacked familiarity with the genetic counseling profession. Even those who expressed some knowledge of the field through prior genetic testing for themselves or a relative did not feel confident in their understanding. When pressed to offer “best guesses” as to what a genetic counselor might do, some interviewees provided basic interpretations based on the profession’s title; “I imagine that they counsel you in genetics [Laughter]” (RNR 03). Others endeavored to provide more detailed guesses:

“I could only assume, by the title—that, if you had a genetic disorder, they be [sic] able to help you understand what it is more and understand the things to watch for or understand what you can do to help mitigate the possibility of somethin’ happening” (RNR 13).

Ultimately, we found that the participants had dichotomous understandings of genetic counseling, emphasizing either the genetics or the counseling aspect of the field. Participants who defined genetic counselors by their genetics expertise perceived them as professionals who could explain family history and risk, explain positive diagnoses, clarify a genetic diagnosis, and recommend lifestyle changes as necessary. Those participants who focused on the counseling component, by contrast, perceived genetic counselors “more as emotional support than clinical support” (RNR 41). Under this interpretation, genetic counselors were viewed as skilled providers who help patients cope with positive diagnoses, support emotional and mental well-being, and attend to the patient’s “psyche” (RNR 41). Regardless of where participants fell in their understanding, they believed genetic counseling support was not relevant to their personal situation, but might be good for others.

### 3.3. Us and Them: Genetic Counseling is for Other People

In the minds of our participants, genetic counseling was better suited for people who are uncertain about study participation, worried about genetic discrimination, not in the medical field, or uneducated in genetics. Genetic counseling was seen as a useful resource for people with a family history of disease or individuals living with a genetic condition. Others perceived as ideal candidates for genetic counseling support included young people, parents, people who are nervous, and people who have difficulty coping.

Our participants maintained that prior to sequencing they were not afraid of what the results could reveal; they were not worriers. They embraced a philosophy that “knowledge is power” (RNR 45), and that it is better to know than not know. In the words of one participant, “Why wouldn’t you want to know?” (RNR 08). This commitment to knowing, coupled with a seemingly unwavering confidence that they “could react properly to whatever was found” (RNR 37), led participants to conclude, “I don’t think I really need to go be counseled by how I’m gonna react if I find out an answer” (RNR 48).

Participant views were also shaped by a strong commitment to research participation: “I decided no matter what was found, you know, I wanted to contribute to the body of knowledge” (RNR 33). The nature of the RAVE study led some participants to conclude that speaking with a genetic counselor prior to undergoing sequencing was unnecessary. They trusted the Mayo Clinic and its biobank, confident that Mayo protects private health information and that “they wouldn’t expose me to anything that would, uh, cause me harm” (RNR 37). Participants perceived the study as “low risk” given that no additional medical procedures were required and felt it was convenient to participate in the study: “I think just contributing to research, and I guess I would add just the fact that it was easy to do. I—[Interviewer: Okay.]—I didn’t have to go into the clinic or get any extra blood drawn or anything” (RNR 05). Ultimately, the participants believed they understood the study aims and procedures well enough to make a decision about their participation: “I, um, have a—enough of a general understanding of, you know the genetics, and […] the results to understand what I was getting into and […] the possibilities of getting, you know, results that weren’t maybe favorable” (RNR 27).

### 3.4. What’s the Value of Speaking with a Genetic Counselor?

Although the participants shared various reasons why they did not need assistance from a genetic counselor, it was clear many questioned the value of such services more generally. They did not know how speaking with a genetic counselor prior to sequencing would benefit them: “I guess I would be interested in exactly what value that would bring to me in terms of my health” (RNR 06). Moreover, the interviewees articulated not knowing what they would discuss in advance of knowing results: “I guess I just wouldn’t know what to ask. I wouldn’t know what to expect or ask or do, really. So—I guess that would be why I wouldn’t do it before I really knew the results” (RNR 09). A few added that they did not want to assume the burden of trying to figure out why they should call: “I guess I didn’t know really what they did and so I guess I wasn’t interested in, you know, finding out anything else about that I guess” (RNR 10). Absent greater familiarity with the value of genetic counseling in advance of pursuing genomic sequencing, the participants felt there was no need to call until they received their results: “Somebody would’ve had to really tell me why it would be a benefit to speak to ‘em first as opposed to doin’ the test first and seein’ if there’s any issues I needed to talk to them about” (RNR 13).

After receiving neutral sequencing results by mail, the participants still did not feel a need to speak with a genetic counselor: “There’s no reason to have to talk to anybody because…there is nothing to talk about. It’s negative” (RNR3). Even if they admitted not completely understanding everything in the results packet, the participants did not feel compelled to call a genetic counselor because the results explicitly stated that nothing was found:

“And the genotype, I have no idea what that is either. But—and it goes all back to the statement that—on the front [of the results letter] that no clinical [sic] significant genetic variants were found. And that’s—that’s the main part. And if one—you know, and if they would’ve even said possible one was a little low, I would’ve called” (RNR 39).

The participants felt they might have been more inclined to talk to a genetic counselor after receiving their results if they had a better sense of how a counselor could help them: “Maybe with more information about […] how I would benefit from having some genetic counseling, I guess. I don’t know” (RNR 26).

Even though nobody we interviewed had reached out to speak with a genetic counselor after receiving their results, 16 out of 50 interviewees posed at least one question to the interviewer that would have been appropriately directed towards a genetic counselor. These questions included study-specific questions (e.g., what genes were tested as part of the study?); questions related to their individual results (e.g., what does the laboratory report mean?); and highly individualized questions regarding their risk in relation to a known family history (e.g., how it is possible to have a family history of a certain disease but the sequencing results did not find anything?).

In spite of a general lack of familiarity with the role or function of genetic counselors, the participants revealed assumptions about what genetic counselors would not be able to do for them, particularly regarding their genomic sequencing results. A few participants assumed that a genetic counselor would not be able to clarify the lab report (at least not in a way that they would be able to understand), explain why they might still be at risk for the diseases in question, or offer any additional genetic health information beyond the letter:

“I feel like the questions I would’ve had, the genetic counselor would not have had the answers to because probably nobody does, you know? [Laughter] It’s probably not known. Like, d—did I have breast cancer markers? So, I mean, they said that they didn’t find anything, variants, so I guess by going and talking to the genetic counselor, I wouldn’t have learned anything more than, you know, what I already know. They’ve already told me that there’s no variants” (RNR 33).

When the interviewers encouraged the participants with questions to reach out to a genetic counselor after the interview, some back-peddled immediately and insisted that getting answers was not important. Others agreed to follow up with a genetic counselor, but none actually did.

## 4. Discussion

Our participants conveyed that unless there was something to talk about, there was no point speaking with a genetic counselor. They maintained that if they understood the benefits and risks of genomic sequencing; were not afraid of receiving results, whatever the outcome; and were not “worriers” by nature, genetic counseling support was unnecessary. For our participants, speaking with a genetic counselor was reserved for receiving a positive result. These findings complement a study by Houfek and colleagues that found that many participants were less keen to discuss, with a genetic counselor, topics related to genetic screening or test decision-making and associated ethical issues, as well as the psychosocial aspects of testing [19].

Although many participants felt that genetic counseling was for others—especially following the receipt of neutral sequencing results—that belief was superimposed on vague, incomplete understandings of the role of genetic counselors. Additionally, the participants did not want to assume the burden of finding out why they should call. This lack of awareness about genetic counseling is not a new phenomenon [13,14], and has been interpreted as a barrier to utilization of genetic counseling services [20]. Inaccurate or incomplete understandings of genetic counseling are particularly problematic in the current climate, where multiple efforts are underway to find effective alternatives to in-person genetic education and counseling. If individuals interested in pursuing genomic sequencing lack a solid grasp of the value of genetic counseling and how they might benefit from such support, they are unlikely to access such services. As we experienced in the RAVE study, even when the participants had questions about some aspect of their results, they were unlikely to reach out to speak with a genetic counselor.

Only 6 out of the 2310 RAVE study participants who received neutral results in the mail elected to contact a genetic counselor for clarification and more information. However, when we interviewed our 50 participants about their experiences receiving neutral results by mail, 32% of our interviewees (*n* = 16) had questions about their results. These experiences are reminiscent of Levin et al.’s findings that genetic counseling uptake increases when counselors are proactive and reach out to patients or research participants directly [11]. Similarly, in a follow-up survey following the return of neutral results, we found that approximately one third of the 1442 study participants who completed the survey had unanswered questions about their results [21].

One might interpret the RAVE study participants’ apparent comfort with unanswered questions about their results as stemming from a place of curiosity, rather than need. However, since a subset of our interviewees misinterpreted their neutral genomic sequencing results (unpublished data), there is arguably a moral imperative to ensure that the recipients of neutral genomic sequencing results have an opportunity to have questions answered and that access to genetic counselors is neither limited solely to individuals who receive medically actionable or especially complicated results nor to those who have a clear understanding of how a genetic counselor can help them. If we know that patients and research participants are less likely to call a genetic counselor directly, more proactive measures may be necessary to facilitate follow-up among individuals who have questions about neutral results. These efforts might include creating educational materials that describe the potential value of genetic counseling support and foregrounding these services more prominently in study materials and results letters. Not surprisingly, individuals’ motivations for accessing genetic counseling services will be different depending on whether they are considering undergoing genomic sequencing or have received sequencing results (see Table 2) and supplementary educational content should reflect those differences. Among the challenges of maximizing scarce genetic counseling resources is identifying, a priori, who needs counseling the most [9]. Pairing elective counseling with supplementary educational content describing the role, scope, and value of genetic counseling services could help individuals with the greatest need self-identify and seek support.

### Study Limitations

Our sample population was very homogenous. Additionally, our biobank participants had previously provided broad consent for future genetic research, which might explain in part their comfort with uncertainty and perceived lack of need for genetic counseling support in the context of the RAVE study. Information about the availability of genetic counseling also was presented in a very neutral way, not in a manner that encouraged utilization. Although the recruitment packet outlined the kinds of questions a genetic counselor could answer, it did not explain the value of accessing genetic counseling services prior to sequencing or after receiving neutral results. Finally, we recruited participants and returned genomic screening results by mail, which may have caused some participants to conclude that follow-up with a genetic specialist was not warranted.

## 5. Conclusions

That so few people wanted to speak to a genetic counselor provides additional support for the current shift away from pre-test counseling towards a more post-results-focused model that prioritizes individuals with medically actionable results. However, that individuals did not appreciate the potential value of genetic counseling at different stages in the evaluation process underscores a need for large sequencing initiatives to consider creating educational materials that describe genetic counseling, the services provided, and why individuals might find speaking with a genetic counselor helpful, irrespective of the type of result received.

Our participants had questions about their results, but did not see answers to those questions as immediately relevant to their health and well-being. While the participants did not take the initiative to call a genetic counselor, when we called them many were eager to have their questions answered, intimating that demand for elective genetic counseling may not align with individuals’ needs. The low uptake of elective genetic counseling suggests that more actively encouraging individuals with questions to access genetic counseling services is unlikely to overwhelm limited genetic counseling resources. To address the lack of familiarity with genetic counseling, a more detailed presentation of the value of the profession should accompany all alternatives to in-person genetic counseling, particularly approaches that require individuals to opt-in to receiving such support. Such efforts are critical in helping those individuals who might benefit from genetic counseling to appreciate their unmet support need and seek access.

## Figures and Tables

**Table 1:** Interview participant demographics *.

	N = 50 **
**Sex**	
Female	25
Male	25
**Age**	
Range	42–71
Mean (SD)	60.9 (6.9)
Race ***	
White	49
Native American	1
**Education**	
High school graduate	5
Some college	18
College graduate	17
Graduate school	8
Other	1
Accessed genetic counseling services during RAVE study	0
Asked the interviewer questions about their neutral results that would have been appropriate to ask of a genetic counselor	16

* Data ascertained from baseline psychosocial survey [16]. ** One participant did not respond to any of the demographic information provided in the survey. *** One participant responded that they were both White and Native American.

**Table 2:** Comparison of the RAVE study participants’ reasons for electing to speak with a genetic counselor before sequencing and after receiving neutral results.

How Many Called?	Why Did They Call?
**8** individuals (out of 5110) invited to participate in the RAVE study called a genetic counselor either prior to or shortly after enrolling.	To establish whether results could have negative implications for acquiring long-term health or life insurance for themselves and/or their children [9] To understand how potential results could effect individual and familial health [9]. To learn about the different types of results they could receive [9]. To find out whether there was a downside to learning some sequencing results. To explore whether they could cope if they received a positive result. To find out the exact genes being sequenced. To make sure that insurance companies would not have access to their genome.
**6** RAVE study participants (out of 2310) who received neutral genomic sequencing results by mail called a genetic counselor.	To discuss family health histories outside the scope of the RAVE study. To confirm that they understood their neutral results. To have their neutral results explained.
